# Integrating allostasis and emerging technologies to study complex diseases

**DOI:** 10.1038/s42003-025-08939-3

**Published:** 2025-11-05

**Authors:** InWha Park, Hyokyeong Gwon, Yeonjeong Jung, Boyoung Kim, Gaeun Ju, Eugene Sin, Hye In An, Hye Jung Bang, Taegwan Yun, Seung Hwan Lee, Wonsik Lee, Choon-Gon Jang, Hyo-Jong Lee, Chung Sub Kim, Jeongmi Lee, Soah Lee

**Affiliations:** 1 School of Pharmacy, Sungkyunkwan University, Suwon, Republic of Korea; 2 Department of Biopharmaceutical Convergence, Sungkyunkwan University, Suwon, Republic of Korea; 3 Department of Pharmacology, School of Pharmacy, Sungkyunkwan University, Suwon, Republic of Korea; 4 Department of Biohealth Regulatory Science, Sungkyunkwan University, Suwon, Republic of Korea

**Keywords:** Complexity, Stem-cell biotechnology, Biological models, Analytical biochemistry, Biomarkers

## Abstract

The study of complex diseases has traditionally relied on reductionist methods, which, although informative, tend to overlook the dynamic interactions and systemic interconnectivity inherent in biological systems. Allostasis, a framework that focuses on physiological adaptations to stress and the maintenance of stability through change, provides a valuable perspective for understanding these diseases. This review summarizes how the allostasis framework defines the cumulative physiological burden—known as allostatic load—imposed by chronic stressors such as persistent psychosocial pressure, drug abuse, and chronic infections. It also explores how adaptive physiological shifts, or changes in allostatic state, contribute to disorders, particularly drug addiction, immune diseases, and cancer. We then review recent studies that uncover stress adaptation mechanisms using cutting-edge technologies, such as multi-omics approaches, induced pluripotent stem cells (iPSCs), and organoid technology. This integrative approach, combining advanced technologies with the allostasis framework, can deepen our understanding of complex disease pathogenesis and inform the development of more effective diagnostic and therapeutic strategies.

## Abstract (web summary)

This review applies the allostasis framework to complex diseases, linking chronic stress and allostatic load to drug addiction, immune diseases, and cancer, and highlights multi-omics, iPSC, and organoid technologies as emerging tools to study them.

## Introduction

**Fig. 1:**
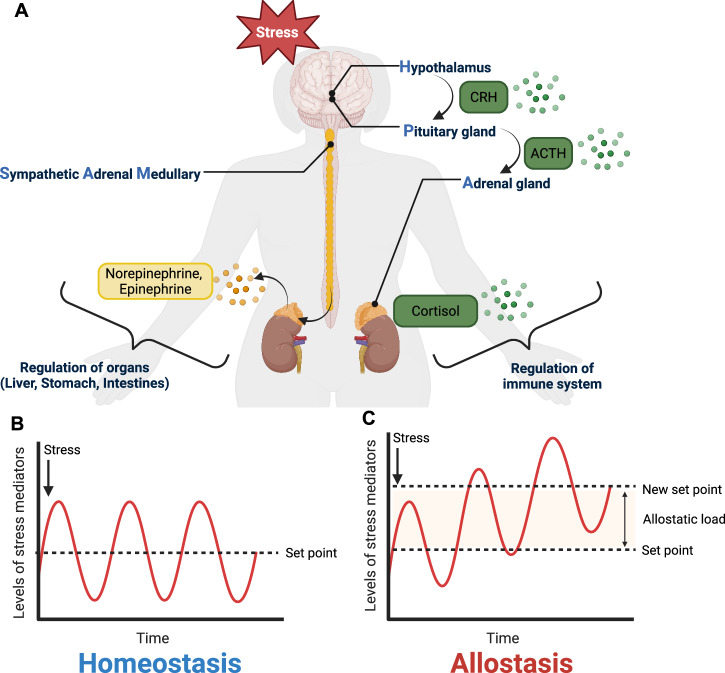
Schematics of biological responses to stress and the concepts of homeostasis and allostasis. **A** Stress-induced activation of the hypothalamic-pituitary-adrenal (HPA) and sympathetic-adrenal-medullary (SAM) axes. The release of catecholamines (e.g., norepinephrine, epinephrine) and glucocorticoids (e.g., cortisol) from both systems regulates the organs, tissues, and immune system. **B**, **C** Homeostasis adapts to stress by maintaining parameters at a constant set point (**B**), while allostasis adapts to stress by adjusting parameters to a new set point (**C**). The difference between the original and new set points represents the cumulative cost of adaptation to stress, or allostatic load. Figure created with BioRender.com.

Throughout life, the human body is exposed to a wide array of physiological and psychological challenges. These range from acute events like physical injury or infection to more insidious, chronic insults that accumulate over time. Among the most significant of these persistent stressors are psychosocial pressures, repeated drug use, and chronic infections. When exposed to these stressors, the body activates its core stress response systems, most notably the neuroendocrine and sympathetic nervous system. These systems operate through key pathways such as the hypothalamic-pituitary-adrenal (HPA) axis and sympathetic-adrenal-medullary (SAM) axis, coordinating the release of hormones like cortisol and adrenaline to initiate the classic “fight or flight” response (Fig. 1A)^1^.

This tightly coordinated response has traditionally been understood through the lens of homeostasis, a concept introduced by Walter Cannon (1871–1945) in 1926. Homeostasis posits that health is maintained by keeping key physiological parameters—such as temperature, blood pressure, and hormone levels—within narrow, stable ranges (Fig. 1B)^2^. Within this view, any deviation from these “set points” is typically seen as pathological. However, this static model falls short in explaining how the body responds to complex and prolonged challenges, such as chronic stressors. For instance, cortisol normally follows a circadian rhythm, peaking in the morning and tapering off by evening. This rhythmic signaling is tightly linked to immune, metabolic, and cardiovascular regulation. Under chronic psychosocial stress, baseline cortisol levels rise and the daily oscillation becomes flattened, disrupting normal system-wide coordination^3^. Such long-term adaptations cannot be explained by a simple return to a fixed set point, underscoring the limitations of the homeostatic framework.

To more accurately reflect the dynamic and integrative nature of physiological responses to stress, Sterling and Eyer introduced the concept of allostasis in 1988 (Fig. 1C)^4^. Allostasis describes how the body achieves stability through change, adjusting physiological set points in response to environmental or internal challenges. This framework recognizes the inter-system coordination required to maintain health and emphasizes that the body often shifts to new equilibrium states, rather than returning to a rigid baseline.

While temporary and dynamic physiological deviations—referred to as the allostatic state—are part of a healthy adaptive process, prolonged or repeated activation of stress response systems can become maladaptive^5^. Chronic stress leads to the accumulation of physiological burden across multiple systems, a burden referred to as allostatic load—the cost of maintaining allostasis over time (Box 1). When this burden exceeds the body’s adaptive capacity, it results in allostatic overload, characterized by systemic dysregulation and an increased risk for disease. Thus, while allostasis is essential for flexible adaptation to acute demands, chronic overuse of the same regulatory pathways contributes to pathogenic shifts and the development of stress-related disorders.

Despite the importance of this framework, most prior allostasis research has focused narrowly on neuropsychological conditions. However, recent studies have begun to broaden this focus, highlighting the role of allostatic load and overload in the development of immune-mediated diseases and cancer. Concurrently, advances in biotechnologies, including multi-omics platforms and iPSC-derived organoid models, are enabling deeper exploration of how chronic stressors, such as sustained neuroendocrine activation and persistent pathogen exposure, contribute to complex disease pathogenesis. This expanding body of work underscores the need for reassessing the current status of allostasis research.

In this review, we examine recent studies applying the allostasis framework to complex diseases, with a focus on neuropsychological disorders, immune diseases, and cancer. We also highlight recent advances in technology-driven models, such as multi-omics analyses and iPSC-based systems, that offer holistic and mechanistic insights into the cumulative burden of chronic stressors on human health.

Box 1 Measurement of allostatic load: clinimetric tools and biomarker-based indices Modern allostasis research utilizes clinimetric tools and biomarker-based indices to capture both clinical and physiological aspects of allostatic load^81–83^.

Clinimetric tools are designed to assess subjective or clinical dimensions of health (e.g., psychosocial stress, depressive symptoms) using structured interviews and questionnaires. Tools such as the Psychosocial Index (PSI), Clinical Interview for Depression (CID), and Diagnostic Criteria for Psychosomatic Research (DCPR)—have significantly advanced allostatic load assessment by complementing biomarker-based evaluation^81,83–85^. Clinimetric assessments help contextualize physiological data by identifying psychological and behavioral contributors to allostatic load, offering clinically meaningful insights for interpreting biomarker-based results.

The allostatic load index is a composite biomarker-based score used to quantify the cumulative physiological burden resulting from chronic stress. It provides a quantitative framework to assess dysregulation across multiple physiological domains, including immune, metabolic, and neuroendocrine axes (Table 1)^33,86^. Common immune biomarkers include C-reactive protein (CRP); metabolic markers include high-density lipoprotein, hemoglobin A1c, and body mass index (BMI); and neuroendocrine biomarkers include cortisol, dehydroepiandrosterone (DHEA), and epinephrine^87^.

A major limitation associated with the allostatic load index in existing studies is the lack of standardized criteria for defining allostatic load. Different studies use varying combinations of biomarkers and thresholds, which limits cross-study comparability and reproducibility. For example, Honkalampi et al. evaluated depressive symptoms using an allostatic load index composed of six cardiovascular and metabolic biomarkers, including systolic and diastolic blood pressure, total cholesterol, waist circumference, BMI, and CRP^10^. In contrast, Osei et al. used a broader panel of 12 biomarkers, spanning neuroendocrine, metabolic, and inflammatory systems, to evaluate depressive disorders, including cortisol, DHEA-S, norepinephrine, epinephrine, HDL, triglycerides, hemoglobin A1c (HbA1c), fasting glucose, CRP, systolic and diastolic blood pressure, and waist-hip ratio^88^. The development of a standardized allostatic load index would facilitate consistent and comparable assessments across various complex disease contexts. Ultimately, the integration of biomarker-based allostatic load index with clinimetric tools provides a comprehensive and multidimensional framework for evaluating allostatic load.

### Allostasis in disease

#### Allostasis in neuropsychological disorders

Activation of the HPA and SAM axes in response to stress leads to the release of cortisol and other stress mediators. While this response is adaptive in the short term, chronic activation of the HPA and SAM axes leads to neuroendocrine dysregulation, thereby increasing the risk of neuropsychological disorders. Although the link between chronic stress and these disorders is well established, most studies have focused on comparing pre- and post-disease states, rather than investigating the intermediate adaptive phases, or allostatic states, that precede disease onset.

One of the most extensively studied conditions within the allostasis framework is drug addiction^6,7^. Research in addiction illustrates how chronic drug use drives the body through a series of dynamic neurobiological transitions—from drug-naive to transition, dependence, and ultimately abstinence—each corresponding to distinct shifts in allostatic state (Box 2)^8^. These intermediate allostatic states provide a mechanistic window into the progressive accumulation of allostatic load that precedes the manifestation of fully developed pathological conditions.

The allostatic load index has been a valuable tool for quantifying stress-related physiological changes and identifying the intermediate allostatic states (Box 1). It has been widely employed in studies investigating the relationship between chronic stress and neuropsychological disorders. For example, individuals with schizophrenia exhibit significantly elevated allostatic load indices than age-matched controls, particularly in neuroendocrine and immune biomarkers^9^. Similarly, patients with depression often show higher allostatic load indices compared to non-depressed controls^10^, along with cortisol levels positively correlating with the severity of depressive symptoms^11^. These findings underscore the value of the allostatic load index as a quantitative marker of chronic stress-related physiological adaptation and allostatic dysregulation.

Beyond its use as a quantitative biomarker, the allostatic load index has broader clinical applications. One study proposed its use for evaluating the effectiveness of pharmacological and psychological interventions in patients with schizophrenia or bipolar disorder^12^. Moreover, therapeutic strategies targeting allostatic biomarkers are being explored as a novel approach for treating neuropsychological disorders^13^. For instance, in individuals with depression who exhibited elevated inflammatory markers—particularly CRP and tumor necrosis factor-alpha (TNF-α)—treatment with infliximab, a TNF-α antagonist, led to improvements in depressive symptoms^14^. This suggests that targeting key allostatic load biomarkers may alleviate allostatic load and offer therapeutic benefit.

Taken together, understanding allostatic states not only provides insight into the body’s dynamic and system-level dysregulation but also guides the development of more effective therapeutic interventions. The allostatic load index, grounded in this framework, offers promising tools for early detection, disease monitoring, and the advancement of personalized treatment strategies.

#### Allostasis in the immune system

Stress is a key driver to allostatic load within the immune system and can modulate various immune components. For example, stress stimulates the proliferation of neutrophils and macrophages and induces the release of pro-inflammatory cytokines and chemokines^15^. Similarly, in the central nervous system, stress activates microglia, leading to the secretion of C-C motif chemokine ligand 2 (CCL2), which activates macrophages and sustains inflammatory responses^16^. However, when allostatic states in the immune system persist over time, they can result in irreversible immune dysregulation and immune system re-programming. In recent studies, chronic unpredictable stress (CUS)–such as sporadic light exposure and food or water deprivation–has been shown to drive differentiation of naïve CD4^+^ and CD8^+^ T-cells toward pro-inflammatory phenotypes. This phenotypic shift is associated with increased production of pro-inflammatory factors, such as interleukin-12 (IL-12) and IL-17^17^. CUS also elevated serum corticosterone–a marker of stress–and serum IL-6 level^18,19^, accompanied by microglia activation and a decrease in TGF-β^17^. These experimental findings are supported by clinical evidence: studies have reported elevated CD4^+^, CD8^+^ T-cell subset population frequency^20^, and increased IL-6 levels^21^ in chronically stressed individuals, linking chronic psychosocial stress to systemic inflammation and immune imbalance. Together, these findings underscore the importance of understanding allostatic load and overload as key factors in modulating stress-related immune dysfunction.

In addition to chronic neuropsychological and environmental stress, the allostatic response of the immune system can be triggered by other stressors, including prolonged infection and chronic diseases such as cancer. For example, Long COVID, a chronic form of COVID-19 emerging from the recent pandemic, exhibits widespread dysregulation of the immune system, as demonstrated by prolonged immune cell activation and increased levels of immune-related factors such as cytokines such as IL-4, interferons (IFNs), and CRP^22^. Similarly, human immunodeficiency virus (HIV) triggers immune activation during the acute phase, as evidenced by the proliferation of CD4⁺ T-cells and elevated levels of inflammatory biomarkers such as IL-6, D-dimer, and CRP^23^. Pro-inflammatory cytokines, including TNF-α, IL-6, and IL-1β also rise^24^, along with activation of the NF-κB pathway^25^. However, in the chronic phase of HIV infection, people living with HIV (PLHIV) exhibit sustained immune dysregulation, characterized by persistent activation of the IL-1β pathway and elevated levels of IL-18 and IL-6^26^. This shift reflects a long-term alteration in the innate immune profile, which is no longer a transient antiviral response but a maladaptive state that contributes to chronic systemic inflammation. Collectively, these immune changes reflect progressive alterations in allostatic states and prolonged innate immune activation, which contribute to chronic infection-associated comorbidities, including cardiovascular disease and neuroinflammation^27,28^.

Chronic diseases such as cancer also impose an allostatic load on the immune system. A recent study reported the infiltration of T lymphocytes and activation of NF-κB and TNF-α pathway in chronic tumor immune microenvironment using multi-omics factor analysis^29^. Within this microenvironment, tumor-associated macrophages and T cells drive the increased production of immune factors such as IFNs, TNF-α, and ILs, which are already recognized as key biomarkers of allostatic load and are incorporated into the immune allostatic load index^30^. In sum, allostasis in chronic diseases and stress conditions leads to elevated levels of immune-related factors, along with broader dysregulation of the immune system.

Despite recent progress, the allostatic response of the immune system remains poorly defined and understudied. As the immune system undergoes dynamic adaptations in response to stressors, including psychosocial stress, chronic infection, and chronic diseases, it is essential to delineate precise molecular and cellular changes underlying immune allostasis. Currently, the allostatic load index includes only a limited set of immune biomarkers, such as CRP, IL-6, and TNF-α^31,32^, highlighting the need to incorporate additional immune allostatic biomarkers such as frequencies of activated T-cell subsets and a broader range of cytokines, to more accurately capture immune allostatic load and overload.

#### Allostasis in cancer

Recent studies have reported that persistent allostatic states driven by chronic stressors are linked to cancer development^33,34^. Allostatic load, specifically characterized in allostatic load index reflecting metabolic and inflammatory dysregulation—such as elevated blood glucose, CRP, or altered lipid levels, has been shown to collectively promote a tumor-permissive environment^35^. Over time, this dysregulation contributes to DNA damage, immune suppression, and chronic inflammation, which are known as hallmarks of tumor initiation, progression, and metastasis^36^.

The cumulative biological impact of chronic stress has been widely studied in cancer patients using the allostatic load index^37,38^. Although promising, the utility of the allostatic load index in predicting cancer development and prognosis remains controversial, with findings varying across populations, cancer types, and assessment methods. For example, one study demonstrated the potential utility of the allostatic load index in predicting long-term health risks among breast cancer patients^39^. In this study, the allostatic load index, calculated by summing the number of high-risk physiological biomarkers, including systolic and diastolic blood pressure, BMI, fasting glucose, total cholesterol, HDL cholesterol, CRP, was found to be elevated in black breast cancer survivors compared to black women without a cancer history, suggesting a possible link between allostatic load and long-term prognosis in this population. Notably, this association was not observed in other racial groups, indicating that the impact of allostatic load may differ by race.

In contrast, some studies have reported no significant association between allostatic load and cancer development. For example, in an elderly Puerto Rican cohort, an allostatic load index based on cardiovascular, metabolic, and inflammatory biomarkers showed no significant correlation with self-reported cancer status, suggesting that the relationship between allostatic load and cancer may vary depending on population characteristics and assessment methods^40^.

These seemingly conflicting results underscore key limitations in current studies investigating allostasis in the context of cancer. Most findings are derived from cross-sectional studies, which hinders causal inference and limit long-term effects of allostatic load on cancer outcomes^41^. Moreover, inconsistent metrics and methodologies, with some studies focusing solely on physiological markers and others incorporating clinimetric tools to assess for psychosocial stress, complicate cross-study comparisons^42^. Finally, previous studies have largely focused on specific types of cancers such as breast and prostate cancers, limiting broader insights across other cancer types.

These limitations emphasize the need for future research on cancer studies with allostasis framework, specifically standardization of allostatic load metrics as well as longitudinal studies across diverse cancer types. This will help clarify the relationship between allostatic load and cancer initiation and progression, and aid in identifying reliable biomarkers for prognosis and treatment. Moreover, molecular experiments using cell- and animal-based models are needed to investigate how chronic stress-induced allostatic state changes mechanistically contributes to tumor initiation and progression. Such studies will help bridge the gap between observational findings and causative biological pathways.

Box 2 Allostasis model of drug addiction Drug addiction has been widely studied within the allostasis framework, which characterizes addiction as a dynamic process encompassing four distinct stages: drug-naive, transition, dependence, and abstinence. Each stage represents a unique allostatic state, marked by progressive dysregulation of neurochemical (e.g., dopamine, opioids, serotonin, gamma-aminobutyric acid) and neuroendocrine (e.g., corticotropin releasing factor, glucocorticoids) systems involved in reward processing and stress responsivity^19^. For example, levels of dopamine, which is the main neurochemical of the brain reward system, gradually increase during drug-naïve to transition states, while significantly decrease in dependence states due to neuroplasticity of the dopaminergic system, which cannot be recovered with abstinence of the drug.

These neuroadaptations contribute to a broader shift in the brain’s reward processing, whereby chronic drug use leads to a lowered ‘hedonic set point.’ As a result, individuals experience reduced pleasure from natural rewards and heightened vulnerability to negative emotional states such as dysphoria and anxiety. This reflects the imbalance between two opposing mood-regulating systems: the reward-facilitating activational process and the counteradaptive mechanism that dampens excessive mood elevation. With continued use, the reward system becomes exhausted, and the stress system—especially the HPA axis—remains overactivated, leading to a new, negatively skewed allostatic set point. During abstinence, the reward system is underactive while the stress system remains elevated, contributing to withdrawal symptoms and relapse risk.

This model highlights the importance of intermediate adaptive states by illustrating stage-specific neurochemical and neuroendocrine changes, thereby providing a molecular basis for understanding stress-induced pathophysiological processes.

## Emerging technologies for advancing allostasis research in complex diseases

### Omics technologies

#### Omics approaches within the allostasis framework

Omics technologies are advanced methods that enable the generation and analysis of large-scale biological data across various types of biomolecules. Major omics platforms such as genomics, transcriptomics, proteomics, and metabolomics, are widely used in systems biology, and a detailed review of their analytical methodologies can be found in the following article^43^. These technologies play a critical role in elucidating complex biological interactions and in uncovering dynamic, system-level responses to external stressors.

**Fig. 2:**
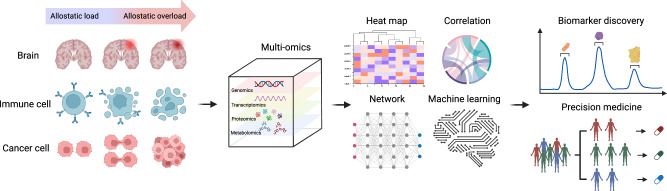
Multi-omics approaches to investigating allostasis in health and disease. This schematic illustrates a brief overview of multi-omics data integration—including genomics, transcriptomics, proteomics, and metabolomics—in the context of disease progression from allostatic load to allostatic overload. It introduces methods based on inter-omics correlation analysis, multi-level molecular network construction, and machine learning. The ultimate goal of such integration is to discover disease biomarkers, elucidate phenotypic spectra and underlying mechanisms, and identify therapeutic targets to advance precision medicine. Figure created with BioRender.com.

Traditional physiological^44^ and biochemical^45^ approaches often focus on a set of biomarkers or single pathways, falling short in capturing the complexity and interconnectivity of entire biological systems. In contrast, omics technologies enable a dynamic, systems-level understanding of biological responses to external stressors by comprehensively analyzing data across multiple molecular layers, including the genome, transcriptome, proteome, and metabolome (Fig. 2). In particular, they have emerged as powerful tools for the identification of novel biomarkers^46,47^, the elucidation of regulatory pathways associated with resilience and vulnerability^48,49^, and the investigation of allostatic load^50^.

Since omics data reflect multilayered and interactive molecular networks, network-based analyses can uncover meaningful patterns and relationships, thereby providing a more comprehensive insight than conventional single-layer approaches^51,52^. Integrating machine learning techniques enables the effective modeling of these complex interactions^53^ and improves the accuracy of disease diagnosis, prediction, and classification^54^. Recently, multimodal strategies that integrate clinical, imaging, and behavioral data with omics information have been effectively applied to predict prognosis and design personalized treatments for complex diseases such as breast cancer^55^.

#### Mechanistic insights into Long COVID-induced allostatic load through multi-omics analyses

Understanding the molecular basis of allostatic regulation requires integrative analytical strategies, particularly in the context of chronic infectious diseases. Long COVID has emerged as a representative condition that imposes a sustained allostatic load, reflecting a state of reduced physiological resilience due to prolonged immune and inflammatory stress.

To further dissect the molecular basis of allostatic burden in Long COVID, multi-omics approaches have proven especially powerful. A recent integrative study combining proteomic, lipidomic, and metabolomic analyses revealed persistent systemic immune responses and identified molecular signatures of sustained macrophage activation, pointing to prolonged immune dysregulation and viral persistence as a key contributor to the chronic condition^56^. More broadly, machine learning applied to transcriptomic and proteomic data has been used to classify immune cell states^57^ and identify neutrophil-related gene clusters for patient stratification^58^. In Long COVID, combined proteomic and metabolomic profiling has facilitated the discovery of candidate biomarkers^59,60^ and enabled prediction of disease severity and mortality^61^. Additionally, multi-layered models that integrate DNA methylation, RNA-seq, proteomic, and metabolomic data have shown high accuracy in predicting both infection status and severity levels^62^.

Thus, the integration of multi-omics and machine learning not only enhances diagnostic and prognostic capabilities for chronic infectious diseases such as Long COVID, but also offers a systems-level framework for probing the molecular underpinnings of allostatic dysregulation.

### Human induced pluripotent stem cell (iPSC)-based models

#### In vitro modeling of allostasis with patient-derived iPSCs

Allostasis research has primarily been conducted using human clinical models and animal models^63^. Clinical studies have primarily assessed allostatic load through biomarker-based indices derived from single time-point measurements^37^. However, factors such as lifestyle variability, environmental influences, cross-sectional study design, and individual genetic differences limit the ability to capture dynamic, individualized molecular responses during allostasis^64^. These limitations highlight the need for personalized and more controlled experimental model to elucidate dynamic regulation of molecular mechanisms underlying allostasis.

**Fig. 3:**
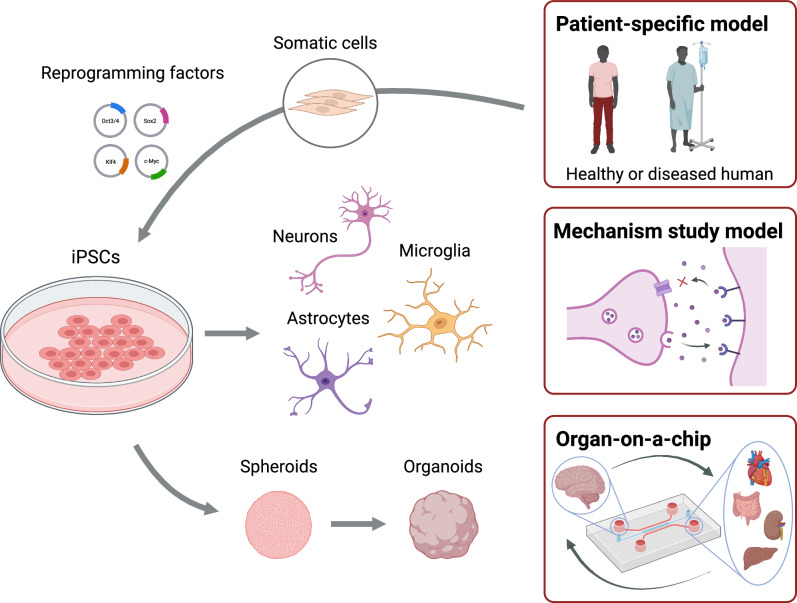
Induced pluripotent stem cell (iPSC) technology for disease modeling and therapeutic research. Somatic cells are reprogrammed into iPSCs using defined factors (Oct4, Sox2, Klf4, c-Myc). These iPSCs can be differentiated into various cell types such as neurons and astrocytes. These cells can be further assembled into 3D structures such as spheroids and organoids. Patient-specific models facilitate mechanistic studies of disease and integration into organ-on-a-chip systems to investigate tissue-tissue interactions and systemic responses relevant to physiological and pathological processes. Figure created with BioRender.com.

Human induced pluripotent stem cells (iPSCs), reprogrammed from a patient’s somatic cells, enable personalized research by capturing the patient’s genetic background while avoiding the ethical concerns associated with embryonic stem cells^65,66^. Human iPSC-based models have emerged as promising tools for studying individual-specific pathophysiological responses with well-controlled environmental factors^67^. These models enable a longitudinal study of biological changes in response to stressors at the molecular and cellular levels, which is not possible in traditional human clinical models (Fig. 3)^68^.

Human iPSC models are increasingly being utilized to study stress-induced neurological diseases, partly due to their accessibility compared to primary cells^69^. These models provide valuable tools for studying allostatic changes in the nervous system in response to prolonged stressor exposure. For example, Cavalleri et al. modeled chronic stress and depression by exposing iPSC-derived dopaminergic neurons to high cortisol levels, observing structural changes and reduced neuroplasticity^70,71^. Similarly, Heard et al. reported that prolonged cortisol exposure to iPSC-derived astropcytes altered in gene expression related to cellular function and morphology, leading to astrocyte dysfunction in both healthy individuals and those with major depressive disorder^72^.

Recent studies have extended the use of human iPSC-based platforms beyond the brain to other organ systems. For example, vascularized liver organoids have been utilized to study metabolic stress responses^73^, while kidney tubule-like cells derived from iPSCs have been used to model nephrotoxic stress response^74^. Cardiac organoids have served as a platform to examine stress-induced cardiac injury and remodeling^75^.

#### iPSC-based multi-organ models for inter-organ allostatic mechanisms

In addition to tissue/organ-specific response to stress, iPSC-based platforms have been utilized to study organ-organ interactions^76^. For instance, Tao et al. developed an iPSC-derived liver-islet multi organ model that recapitulates hyperglycemia-induced metabolic dysfunction and showed that metformin treatment partially restored these phenotypes^77^. Furthermore, iPSC-based platforms have been employed to investigate systemic stress responses to SARS-CoV-2 infection, providing mechanistic insights into inter-organ crosstalk. One study integrated transcriptomic data from SARS-CoV-2 infected iPSC-derived cardiomyocytes and patient blood samples, revealing cytokine-driven feedback loops between infected tissues and circulating immune cells. These findings highlight organ–organ interaction as a key feature of systemic disease progression^78,79^. In a separate study, SARS-CoV-2 elicited conserved inflammatory signaling across multiple iPSC-derived organoids including lung, brain, heart, kidney and liver, suggesting the presence of shared immune responses across diverse tissues^80^. Collectively, these studies underscore the potential of iPSC-based systems to explore allostatic mechanisms across multiple tissues in a patient-specific manner.

## Conclusions and future perspectives

The concept of allostasis provides a dynamic framework for understanding how chronic stressors contribute to disease development through progressive physiological adaptation and dysregulation. While traditionally applied to neuropsychological disorders such as addiction and depression, recent advances have expanded its relevance to immune-mediated diseases, chronic infections, and cancer. These conditions demonstrate that sustained stress responses across neuroendocrine, immune, and metabolic systems can drive allostatic load and, when prolonged, lead to allostatic overload—resulting in multisystem dysfunction.

Despite this conceptual advancement, several gaps remain in both mechanistic understanding and clinical application. Most notably, the measurement of allostatic load lacks standardization. Biomarker panels vary significantly between studies, limiting reproducibility and cross-contextual comparisons. The inclusion of only a narrow subset of immune biomarkers (e.g., CRP, IL-6, TNF-α) in current indices does not fully capture the complexity of immune allostasis, particularly in chronic conditions like Long COVID, HIV, or cancer.

Emerging technologies offer promising solutions. Multi-omics approaches have enabled system-level mapping of allostatic burden across diverse molecular layers, revealing sustained immune activation and metabolic disruption in conditions such as Long COVID. Machine learning further enhances the predictive power of omics data, allowing for individualized disease classification and outcome prediction. Similarly, patient-derived iPSC models provide unprecedented opportunities to study molecular adaptation in response to stressors within specific tissues and across organ systems. These models allow for controlled investigation of allostatic responses that cannot be easily captured in traditional clinical or animal studies.

However, technical limitations still remain. In vitro models often lack full cellular heterogeneity, immune components, and systemic interactions critical for understanding allostasis as an integrative, brain-to-body phenomenon. Therefore, while these tools cannot wholly replicate the complex regulatory dynamics of living organisms, they serve as valuable platforms for dissecting localized mechanisms of stress response, including receptor signaling, gene regulation, and cellular adaptation.

Future research should prioritize: (1) standardizing allostatic load assessment via consensus biomarker panels, (2) conducting longitudinal studies to link chronic stress with allostatic dysregulation, and (3) leveraging integrative platforms like multi-omics and iPSC-based systems to model systemic adaptation. Addressing these gaps within the allostasis framework will enable more precise diagnostics, targeted interventions, and personalized strategies for managing complex, stress-related conditions.

**Table 1:** Biomarkers used to measure allostatic load in neuropsychological disorder, immune disorder, and cancer

	Systems	Disorders		
		Neuropsychology	Immune	Cancer
Biomarkers	Neuroendocrine	Cortisol Dopamine Serotonin GABA DHEA-S Epinephrine Norepinephrine	—	—
	Immune	CRP TNF-α	CRP TNF-α IL-6	CRP
	Metabolic	Fasting glucose Total cholesterol HDL BMI Waist circumference Waist-hip ratio Triglycerides HbA1c	—	Fasting glucose Total cholesterol HDL BMI
	Cardiovascular	Systolic blood pressure Diastolic blood pressure	—	Systolic blood pressure Diastolic blood pressure

*GABA* γ-aminobutyric acid, *DHEA* dehydroepiandrosterone sulfate, *CRP* C-reactive protein, *TNF-α* tumor necrosis factor-α, *HDL* High-density lipoprotein cholesterol, *BMI* Body Mass Index, *HbA1c* Hemoglobin A1c, *IL-6* interleukin-6.
